# Role of Pretreatment Hemoglobin-to-Platelet Ratio in Predicting Survival Outcome of Locally Advanced Nasopharyngeal Carcinoma Patients

**DOI:** 10.1155/2021/1103631

**Published:** 2021-10-30

**Authors:** Cosphiadi Irawan, Andhika Rachman, Puji Rahman, Arif Mansjoer

**Affiliations:** ^1^Division of Hematology and Medical Oncology, Department of Internal Medicine, Dr. Cipto Mangunkusumo National General Hospital–Faculty of Medicine Universitas Indonesia, Jakarta, Indonesia; ^2^Department of Internal Medicine, Dr. Cipto Mangunkusumo National General Hospital–Faculty of Medicine Universitas Indonesia, Jakarta, Indonesia; ^3^Clinical Epidemiology Unit, Department of Internal Medicine, Dr. Cipto Mangunkusumo National General Hospital–Faculty of Medicine Universitas Indonesia, Jakarta, Indonesia

## Abstract

**Background:**

The three-year survival rate of locally advanced nasopharyngeal carcinoma (NPC) patients in Indonesia is lower than in other Asian countries. Calculation of hemoglobin-to-platelet ratio (HPR) may become a more practical predictor than the ratios using leukocyte cell components. Yet, no study has been conducted to investigate the potential of HPR in predicting survival outcomes in locally advanced nasopharyngeal cancer patients.

**Objective:**

To determine the role of pretreatment hemoglobin-to-platelet ratio in predicting the three-year overall survival (OS) of locally advanced NPC.

**Method:**

A retrospective cohort study followed up on 289 locally advanced NPC patients who had undergone therapy at the Dr. Cipto Mangunkusumo National General Hospital between January 2012 and October 2016. HPR cut-off was determined using ROC. Subjects were classified into two groups according to the HPR value. Kaplan-Meier curve was utilized to illustrate patients' three-year survival, and Cox regression test analyzed confounding variables to yield an adjusted hazard ratio (HR).

**Results:**

The optimal cut-off for HPR was 0.362 (AUC 0.6228, 95% CI: 0.56-0.69, sensitivity 61.27%, specificity 60.34%). Of the subjects, 48.44% had HPR ≤ 0.362, and they had a higher three-year mortality rate than those with HPR > 0.362 (50% vs. 31.54%). In bivariate analysis, HPR ≤ 0.362 and age ≥ 60 significantly showed a worse three-year OS (*p* value = 0.003 and 0.075, respectively). In multivariate analysis, we concluded that a pretreatment HPR ≤ 0.362 was an independent negative predictor of three-year OS in locally advanced NPC patients (adjusted HR 1.82; 95% CI: 1.25–2.65).

**Conclusion:**

Pretreatment HPR ≤ 0.362 was a negative predictor of three-year OS in locally advanced nasopharyngeal cancer patients.

## 1. Introduction

Nasopharyngeal carcinoma (NPC) is one of the most common cancer in Indonesia, which has the second-highest global incidence. It is the sixth most common cause of cancer-associated death in Indonesia [1–3]. The three-year survival rate of locally advanced NPC patients in Indonesia is lower than that in other Asian countries [4–6]. A previous study found that in our country, many patients are younger than thirty years old and have high latent membrane protein (LMP1) expression [1]. This fact is associated with high Epstein-Barr virus virulence [1]. Therefore, evaluations of Epstein-Barr virus (EBV) markers at diagnosis and surveillance are recommended as useful predictors for the outcome; however, their application has been limited in Indonesia due to unavailability of the assay [7–10].

The development of other predictors has also been widely studied, one of which functions through the inflammatory pathway. Inflammation is an important factor in cancer progression, and this is reflected in EBV-encoded LMP1 [11, 12]. The stimulatory effect of inflammation may affect peripheral blood cells. Studies have developed predictors utilizing the ratio between peripheral blood cell components, which can be obtained easily using readily available values. These ratios have been reported to be statistically significant in evaluating NPC survival and have been assessed in follow-ups and showed good applicability even in suboptimal conditions [13–15]. However, most of the ratio numerators and denominators involve the numbers obtained from the leukocyte differential count. These numbers may fluctuate in the presence of infection, for example, due to monocytes. In addition, leukocyte cells are viable for a short time only therefore discrepancies between measurements can occur [16].

The calculation of hemoglobin-to-platelet ratio as a single variable may serve as a more reliable predictor than the ratios between leukocyte cell components. This simple measurement has been reported to be useful in predicting outcomes such as disease progression and mortality in bladder cancer [17, 18]. Moreover, in rectal cancer, in addition of being able to predict disease progression, HPR can also help to differentiate between rectal cancer and benign rectal diseases [17, 19]. However, there has been no study conducted to confirm the role of HPR in predicting three-year survival in locally advanced nasopharyngeal cancer patients. The objective of this study was to determine the role of pretreatment hemoglobin-to-platelet ratio (HPR) in predicting the three-year overall survival (OS) of locally advanced NPC. We hypothesized that low pretreatment HPR would result in lower three-year overall survival (OS) of locally advanced NPC.

## 2. Methods

The design of this study was a retrospective cohort using survival analysis. Subjects were locally advanced NPC patients who had undergone therapy at Dr. Cipto Mangunkusumo National General Hospital, Jakarta, Indonesia, between January 2012 and October 2016. Inclusion criteria were as follows:

1. Diagnosis of NPC by histopathology following World Health Organization (WHO) classification and Union for International Cancer Control (UICC)/American Joint Committee on Cancer (AJCC) 7^th^ edition
2. The Eastern Cooperative Oncology Group (ECOG) Performance Scale ≤ 2
3. Completion of all chemo- and radiation therapy cycles

Exclusion criteria were patients with prior and secondary malignancy, double primary malignancies, concurrent infections (hepatitis B/C, AIDS), autoimmune disease, liver cirrhosis, diabetes mellitus, active bleeding signs, coronary heart disease, chronic kidney disease (eGFR <30 ml/min/1,73m^2^), chronic heart failure (NYHA functional class > II), administration of anti-thrombotic and erythropoietic agents, and did not finish chemoradiation therapy. No patients in the study received transfusion of platelets or red blood cells.

Demographic and clinical characteristic data were collected, along with laboratory and comorbidity data. All subjects underwent three-year surveillance to assess for mortality. The study was approved by the Ethics and Research Committee of the Faculty of Medicine, Universitas Indonesia (number: KET-\033/UN2.F1/ETIK/PPM.00.02/2019). Data on the mortality of the study sample were obtained from hospital medical records. If the patient did not come to the hospital for routine posttreatment evaluation, data regarding survival were obtained from the telephone call, home visit or death certificates from the Civil Registry Service Office.

The optimal cut-off value for HPR was determined using the receiver operating characteristic (ROC) curve, and subjects were then classified into two groups, one with HPR ≤ 0.362 and the other with HPR > 0.362. Death within three-year posttherapy was noted, as was survival for three-year posttherapy. The proportional hazard test was carried out on the variables, and pretherapy confounding variables were assessed (*age* ≥ 60, clinical stage, gender, and nutritional status). Variables fulfilling the proportional hazard assumption were included in the bivariate analysis. If variables had a *p* value of <0.25, they were included in the multivariate test using the Cox regression test. Data processing was completed with STATA 15.0.

## 3. Results

In this study, data were collected for 355 subjects, including the pretreatment laboratory analysis. After exclusion criteria were applied, 289 subjects remained, 92.7% of whom were successfully followed up over the three years. The baseline characteristics of these patients are listed in Table 1. Based on their nutritional status, 16.95% of subjects had BMI<18.5, 42.91% had BMI 18.5–24.9, and 40.13% had BMI > 25. In this study, 40.13% of the subjects had experienced anemia, 58.13% had received conventional radiotherapy, and 37.02% had been irradiated with intensity-modulated radiation therapy (IMRT).

**Figure 1:**
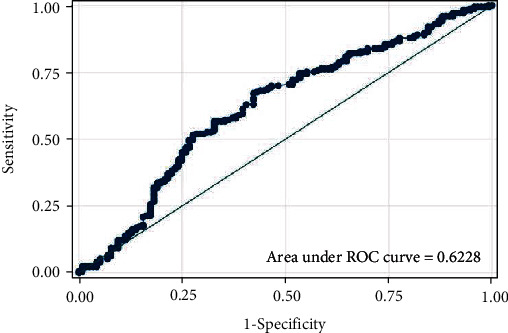
Receiver operating characteristic curve for HPR to three-year mortality (AUC 0.6228, 95% CI: 0.56-0.69).

The optimal cut-off value for HPR was 0.362 (AUC 0.6228, 95% CI: 0.56–0.69, sensitivity 61.27%, specificity 60.34%). The ROC curve for HPR relative to three-year mortality rate is illustrated in Figure 1. The optimal cut-off for hemoglobin was 129 g/l (AUC 0.642 (sensitivity 60.12%, specificity 59.48%), whereas for platelets it was 343 × 10^3^/*μ*l (AUC 0.58 (sensitivity 59.48%, specificity 58.96%).

Samples were classified into two groups, one with HPR > 0.362, the other with HPR ≤ 0.362. There were 149 patients (51.56%) in the higher HPR group and 140 patients (48.44%) in the lower HPR group. The lower HPR group had a higher three-year mortality rate than those with the higher HPR groups (50% vs. 31.54%, respectively). The proportion of patients with low BMI, greater weight loss, lower ECOG Performance Scale, and higher stages was greater in the group with low HPR. The baseline characteristics of these two groups are listed in Table 2.

**Figure 2:**
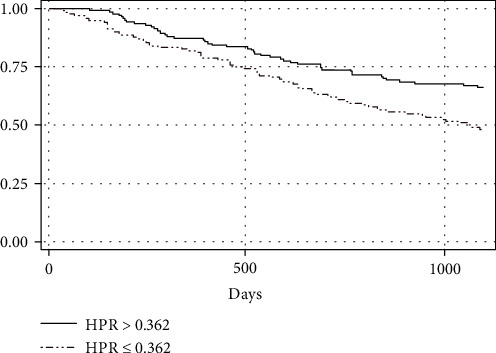
Kaplan-Meier plot comparing three-year OS of locally advanced NPC patients with the HPR group before therapy.

The three-year OS curve showing the difference between the high and low HPR groups can be seen in Figure 2. HPR variable ≤ 0.362 and age ≥ 60 years fulfilled the proportional hazard assumption and were included in the Cox regression test. The bivariate test in Table 3 demonstrated that HPR ≤ 0.362 was the only significant factor influencing the three-year survival with a crude hazard ratio (HR) value of 1.75 (95% CI 1.2–2.25). Multivariate analysis with confounding variables resulted in an adjusted HR HPR value ≤ 0.362 of 1.82 (95% CI 1.25-2.65), as shown in Table 3. In the additional analysis, when used as a separate variable according to the cut-off value, the adjusted HR for hemoglobin ≤ 129 was 1.55 (95% CI 1.04-2.29) and adjusted HR for platelet ≥ 343 × 10^3^ was 1.5 (95% CI 1.02–2.24).

## 4. Discussion

Variations in geographical and ethnic distribution have given rise to NPC being more prevalent in one area and more infrequent in other areas [20]. In our study, the mean age of the subjects was 45.6 years, which is similar to studies conducted in China [4] but differs from other studies conducted in the USA [21]. Exposure to carcinogenic materials at a younger age and better programs for early detection in areas with a high incidence has shifted the peak incidence for NPC to a younger age [1, 20, 22]. The most common clinical stage in our sample was IVA (53.98%). These results differ from an earlier study in Jakarta that showed the most common stage to be IVB (32.7%) [6]. Studies in Hong Kong by Lee et al. and Turkey by Topkan et al. have reported the most prevalent stage to be III (68.24% and 56.4%) [4, 23]. This means that early detection, patient awareness, and access to treatment in Indonesia are better than in the previous decade. The histopathological characteristics of patients in Indonesia are the same as those in China [4] (90% WHO type III), which is chemoradiosensitive, but differ in terms of survival. In this study, the three-year mortality rate was 40.14%, which is better than in earlier studies conducted in Jakarta [6] and Yogyakarta [5] at which time, the rates were 47.1% and 70%, respectively. A study in China revealed a mortality rate of 22% [4]. Differences in the proportions of tumor stages, radiation techniques used, and overall treatment time may make it challenging to compare survival rates.

Furthermore, differences in survival are perhaps to be expected due to genetic variations in the latent membrane protein 1 (LMP1). Adham et al. [1] found that higher EBV-encoded LMP1 expression was seen in patients younger than thirty years old. Overexpression of LMP1 is thought to activate the inflammatory pathway, eventually affecting peripheral blood cells, including hemoglobin, leukocytes, and platelets. The interaction between inflammation and peripheral blood cells within the tumor microenvironment signifies cancer activity [11]. This relationship in NPC is supported by a study by Al-Kholy et al. [24] who demonstrated an association between high pretreatment levels of EBV-DNA and IL-6 with poor survival in locally advanced NPC. The appearance of a peripheral blood smear that has been influenced by IL-6 activation within the tumor microenvironment may predict the degree of inflammation, which in turn affects tumor progression and survival.

Within the tumor microenvironment, progenitor cells activated by inflammation trigger excessive expression of the GATA2 transcription factor, inhibiting erythroid differentiation and raising the megakaryocyte differentiation rate. This occurrence eventually leads to anemia and thrombocytosis [25]. These two conditions work in synergy to cause tumor cell hypoxia and activate the secretion of growth factors and cytokines. In the tumor microenvironment, the increased platelets and the presence of cancer-associated anemia can induce angiogenesis by stimulating vascular endothelial growth factor (VEGF) and upregulating hypoxia inducible factor-1*α* (HIF-1*α*), which has significant role in cancer progression. In addition to angiogenesis, HIF-1*α* upregulation inhibits apoptosis and thus affects cellular survival [18, 23, 26, 27]. This theory has been supported by Wang et al. [28] who concluded that the high expression of GATA-2 in NPC patients is associated with a higher pathologic grade, larger tumor size, and worse prognosis. Hence, a growing body of research suggests the importance of cancer-associated anemia and thrombocytosis in tumor progression and the relationship between the hematologic biomarkers and cancer prognosis.

The use of HPR as a single variable in predicting survival has not been widely studied [17, 18]. This study explored the possibility of using HPR as a prognostic marker in locally advanced NPC patients. The group with lower HPR had higher risks for three-year mortality, even after adjusting for confounders. The role of HPR as a predictor for survival in this study was consistent with previous studies. Tang et al. reported that low HPR (<0.615) were correlated with poor overall survival in stage 1 and grade 3 (T1G3) bladder cancer (HR 1.23, *p* value = 0.003) [18]. Croce et al. found that patient with low HPR had higher risk of cancer-specific mortality and overall mortality, and Mo et al. found that low HPR is associated with high tumor invasion (T stage) and lymph node metastasis in rectal cancer [17, 19]. In colon cancer, decreased HPR was associated with tumor invasion and tumor size [29]. These findings in other types of cancer support our findings in the NPC population, in which similar studies have not been published widely.

To ensure that the HPR values of our subjects were not influenced by external factors, we clarify that no patients in the study received pretreatment transfusion of platelets or RBC. In this study, we lacked median survival data because observations were limited to three years only. Rahajeng et al. [6] reported that the median survival of locally advanced stage NPC patients might be up to 4.6 years.

We found that old age was not an influential factor in a low three-year survival rate, whereas Huang et al. [30] found that age might suppress the five-year survival in NPC patients older than sixty (adjusted HR = 2.42, *p* value ≤ 0.001). The discrepancy between these two research results may have resulted from the number of elderly subjects (34.7%), their multiethnicity, and their age grouping into three groups.

After adjusting for the age factor, the HRs for hemoglobin and platelets were lower than that of the HPR (1.55, 1.5, and 1.82, respectively). These numbers confirm that HPR alone is a more effective predictor. However, HPR should not be used as a sole predictor, but the value it adds to the other prognostic markers in NPC patients should be further investigated.

The strengths of our study lie in a large number of subjects in younger age, the low rate of loss to follow-up (8.3%), and this study being the first to assess the relationship between HPR and survival in locally advanced NPC patients. Our results complement other studies that have proven the relationship between the ratio of other peripheral blood cells and NPC patient survival. Compared to the ratio across absolute leukocyte numbers, HPR is more definitive because acute infections relatively unburden it. HPR calculation is also a simple, inexpensive, and practical for hospitals with suboptimal facilities. In addition, this study analyzed the probability of confounders in the association between HPR and mortality. Thus, a further assessment may be necessary.

Nevertheless, there were possibilities of missing data and information bias. We also excluded diseases that may directly affect the hemoglobin and platelet populations. Regrettably, we did not investigate all underlying factors in patients' mortality.

## 5. Conclusion

A pretreatment HPR of ≤0.362 was a negative predictor of three-year OS in locally advanced nasopharyngeal cancer patients.

## Figures and Tables

**Table 1:** Baseline characteristics of the patients in the study.

Variable	Total (*N* = 289)
Age, year (mean, SD)	45.6 (12.2)
Age group, *n* (%)	
≥60	35 (12.11)
<60	254 (87.99)
Gender, *n* (%)	
Male	204 (70.59)
Female	85 (29.41)
Weight loss, *n* (%)	
0–5 kg	221 (76.47)
5–10 kg	43 (14.88)
>10 kg	25 (8.65)
Smoking history, *n* (%)	
Yes	138 (47.75)
No	151 (52.25)
ECOG performance scale, *n* (%)	
0	40 (13.84)
1	238 (82.35)
2	11 (3.81)
WHO histopathologic grade, *n* (%)	
1	2 (0.69)
2	14 (4.84)
2–3	9 (3.11)
3	261 (90.31)
Unknown	3 (1.04)
Clinical stage, *n* (%)	
III	45(15.57)
IVA	156 (53.98)
IVB	88 (30.45)
T, *n* (%)	
T1-T3	74 (25.61)
T4	215 (74.39)
N, *n* (%)	
N0	25 (8.65)
N1	44 (15.22)
N2	131 (45.33)
N3a	131 (45.33)
N3b	64 (22.15)
Therapy, *n* (%)	25 (8.65)
CCRT	201 (69.55)
NAC+CCRT	82 (28.37)
RT	6 (2.08)
RT cycle (fraction (median, min-max))	35 (15-39)
Total dose (Gy (median, min-max))	70 (30-78)
Overall time (days (median, min-max))	52 (23-97)
Chemosensitizer, *n* (%)	
<5 cycles	104 (35.99)
≥5 cycles	185 (64.01)
RT technique, *n* (%)	
2D/3DRCT	168 (58.13)
2D/3DRCT+IMRT	14 (4.84)
IMRT	104 (37.02)
Hemoglobin (g/l (median, min-max))	129 (68-167)
Platelet (×10^3/^*μ*l (median, min-max))	338 (113-801)
Transfusion on CCRT, *n* (%)	
Yes	124 (42.91)
No	165 (57.09)
3-year mortality, *n* (%)	
Yes	116 (40.14%)
No	149 (51.56%)
Lost to follow-up	24 (8.3%)

Notes: 2D: two dimensional; 3D-CRT: three-dimensional-conformal radiotherapy; CCRT: concurrent chemoradiotherapy; ECOG: Eastern Cooperative Oncology Group; IMRT: intensity-modulated radiotherapy; NAC: neoadjuvant chemotherapy; RT: radiotherapy; WHO: World Health Organization.

**Table 2:** Comparison of baseline characteristics across the HPR groups.

Variable	HPR > 0.362 (*N* = 149)	HPR ≤ 0.362 (*N* = 140)
Age group, *n* (%)		
≥60	23 (15.44)	12 (8.57)
<60	126 (84.56)	128 (91.43)
Gender, *n* (%)		
Male	117 (78.52)	87 (62.14)
Female	32 (21.48)	53 (37.86)
BMI (kg/m^2^ median, min-max)	23.04 (13.67-37.47)	21.39 (13.34-35.34)
Weight loss, *n* (%)		
0-5 kg	127 (85.23)	94 (67.14)
5-10 kg	13 (8.72)	30 (21.43)
>10 kg	9 (6.04)	16 (11.43)
Smoking history, *n* (%)		
Yes	76 (51.01)	62 (44.29)
No	73 (48.99)	78 (55.71)
ECOG performance scale, *n* (%)		
0	25 (16.78)	15 (10.71)
1	122 (81.88)	116 (82.86)
2	2 (1.34)	9 (6.43)
WHO histopathologic grade, *n* (%)		
1	1 (0.67)	1 (0.71)
2	7 (4.7)	7 (5)
2-3	6 (4.03)	3 (2.14)
3	133 (89.26)	128 (91.43)
Unknown	2 (1.34)	1 (0.71)
Clinical stage, *n* (%)		
III	30 (20.13)	15 (10.71)
IVA	78 (52.35)	78 (55.71)
IVB	41 (27.52)	47 (33.58)
T, *n* (%)		
T1-T3	45 (30.2)	74 (25.61)
T4	104 (69.8)	215 (74.39)
N, *n* (%)		
N0-N1	40 (26.85)	29 (20.71)
N2-N3	109 (73.15)	111 (79.29)
Therapy, *n* (%)		
CCRT	110 (73.83)	91 (65)
NAC+CCRT	35 (23.49)	47 (33.57)
RT	4 (2.68)	2 (1.43)
RT cycle, fraction (median, min-max)	34.46 (33-39)	34.23 (15-39)
Total dose (Gy (median, min-max))	69.69 (66-78)	68.73 (30-78)
Overall time (days (median, min-max))	54.68 (40-91)	56.41(23-97)
Chemosensitizer, *n* (%)		
<5 cycles	54 (36.24)	50 (35.71)
≥5 cycles	95 (63.76)	90 (64.29)
RT technique, *n* (%)		
2D/3DRCT	85 (57.05)	83 (59.29)
2D/3DRCT+IMRT	8 (5.37)	6 (4.29)
IMRT	56 (37.58)	51 (36.42)
Transfusion on CCRT, *n* (%)		
Yes	49 (32.89)	75 (53.57)
No	100 (67.11)	65 (46.43)
Follow up duration (days (median, min-max))	822 (65-1095)	760 (27-1095)

Note: 2D: two dimensional; 3D-CRT: three-dimensional-conformal radiotherapy; BMI: body mass index; CCRT: concurrent chemoradiotherapy; ECOG: Eastern Cooperative Oncology Group; IMRT: intensity-modulated radiotherapy; NAC: neoadjuvant chemotherapy; RT: radiotherapy; WHO: World Health Organization.

**Table 3:** HPR and age as predictors of survival at three years; crude and adjusted HR for confounding variable with HPR ≤ 0.362.

Variable	Person Time	Failure	Incidence Rate	Hazard Ratio	*p*
HPR				1.75	0.003
≤0.362	106479	70	0.00037541		
>0.362	122532	46	0.00065741		
Age group				1.58	0.075
≥60	23836	18	0.00075516		
*<60*	205175	118	0.00047764		
Crude HR					
HPR ≤ 0.362				1.75 (1.2–2.55)	
Adjusted HR					
+old age				1.82 (1.25–2.65)	

Note: HPR: hemoglobin to platelet ratio. HR: Hazard ratio.

## Data Availability

Additional data can be acquired by contacting the corresponding author using the email address provided.

## Additional Points

Pretreatment hemoglobin-to-platelet ratio (HPR) of ≤0.362 is a negative predictor of three-year overall survival in locally advanced nasopharyngeal cancer patients.
